# Evaluation of Histological Impacts of Three Types of Orthodontic Fixed Retainers on Periodontium of Rabbits

**Published:** 2014-09

**Authors:** Morteza Oshagh, Somayeh Heidary, Ali Dehghani Nazhvani, Fatemeh Koohpeima, Omid Koohi Hosseinabadi

**Affiliations:** a Orthodontist, Private Practice, Tehran, Iran.; b Orthodontist, Student Research Committee, Shiraz University of Medical Sciences, Shiraz, Iran., Dept. of Orthodontics, School of Dentistry, Bushehr University of Medical Sciences, Bushehr, Iran.; c Dept. of Oral & Maxillofacial Pathology, Biomaterial Research Center, School of Dentistry, Shiraz University of Medical Sciences, Shiraz, Iran.; d Dept. of Operative Dentistry, School of Dentistry, Shiraz University of Medical Sciences, Shiraz, Iran.; e Stem cell and Transgenic Technology Research Center, Shiraz University of Medical Sciences, Shiraz, Iran.

**Keywords:** Fixed orthodontic retainers, Periodontium, Histology, Rabbits

## Abstract

**Statement of the Problem:** Fixed retainers were developed to maintain incisor alignments after orthodontic treatments. Although the effects of fixed retainers on periodontal health are clinically studied, no studies have still evaluated the histological changes in the periodontium after the placement of thefixed retainers.

**Purpose: **The aim of this study was to evaluate the effects of customised retainers on periodontium histologically.

**Materials and Method: **Forty pairs of maxillary and mandibular central incisors of twenty rabbits were randomly divided into four equal groups: The first group was considered as the control and in the second group, Fiber Reinforced Composite (FRC), in the third group, 0.014 inch stainless steel (SS) wire and in the fourth group, 0.175 inch multistrand stainless steel (MSS) wire were bonded on the labial surfaces of the incisors. After sixty days; animals' periodontium were evaluated histologically.

**Results:** The number of bone resorption lacuna in the control group was significantly less than FRC and 0.014 SS groups. The periodontal vessel count and their diameter in the control group was significantly lower than the other groups. The pulp vessel count and their diameter in controls were significantly more than the 0.014 SS and the 0.175 MSS groups.

**Conclusion:** Findings of this study suggest that FRC fixed retainer might cause detrimental effects on the periodontal ligaments and supporting bone and the 0.014- inch and 0.175- inch fixed retainers can cause hyalinization and possibly the necrosis of the pulp.

## Introduction

One of the major challenges of the orthodontists is the long-term stability of the orthodontic treatments. This has elicited the orthodontists to seek methods to ensure the corrections made during treatment to remain stable. Different investigators have suggested treatment methods that can enhance the stability of treatment, and this has led to developing removable and fixed retainers [1-8]. Many orthodontists believe that the only way to maintain ideal alignment after treatment is some form of permanent retention [8-10], therefore, fixed bonded retainers now are being left in the mouth for long period of time [8]. The major advantage of the bonded retainers, compared to to the removables, is that they are compliant free, except for their difficult oral hygiene maintenance [11].

Fixed retainers were first introduced in 1970s [12] and remain a vital component of orthodontic treatment. The first generation consisted of a large diameter stainless steel (SS) round wire bonded only to the lingual surface of the canines [13]. Later, Zachrisson introduced the use of co-axial or braided wires in small diameter bonded to all mandibular anterior teeth [14-15]. The use of multi-stranded wires for the construction of the fixed retainers has been proposed based on their ability to allow the physiological movement of the teeth. Whereas their braided surface offered increased mechanical retention with the adhesive bondings [14-16].

Glass fiber-reinforced composite resins (FRC) were developed for restorative purposes [17-18] and later found their way into orthodontics as a retention splint [18-20]. One main advantage of fiber-reinforced resin composites is their biocompatibility, especially in patients who are allergic to nickel, present in the stainless steel wires [21]. Another advantage is their favorite esthetics, since no metal wire is present in the structure of fiber-reinforced composites [22]. These retainers create a rigid splint which might contribute to a limited physiological tooth movement [23-25]. Whenever any new product is introduced in medicine, or when a supplementary indication for its usage is described, it would be compared to other custom products or standards. Multi-stranded wire retainers are widely accepted and are considered as a standard treatment option in modern orthodontics [21].

Although several factors contribute to the development of gingival recession [26], periodontal disease and mechanical trauma are the two primary etiological factors in the pathogenesis of gingival recessions [26-29]. Fixed retainers, in general, have been criticized for their potential in compromising the periodontal health. The mode of functional loads that are exerted on anterior teeth changes following the splinting with the fixed retainers, which in turn compromises the health of periodontium. However; the studies regarding the consequences of splinting on the status of periodontium are limited [8, 30-31].

There is still Controversy over the presence of a bonded lingual retainer to have a negative effect on the periodontal tissues [32]. Even though the effect of different orthodontic fixed retainer on periodontal status has been studied in previous investigations [8, 11, 13, 16], to the best of our knowledge, no studies have still evaluated the histological changes in the periodontium following the placement of different fixed retainers. Therefore, the aim of this study was to evaluate and to compare the histological effects of customized retainers with wire diameters of 0.014 inch stainless steel and 0.175 inch multistrand stainless steel wire as well as the fiber-reinforced composite retainer on periodontal health.

## Materials and Method

**Animals**

This experimental study was performed on 20 white male New Zealander rabbits; each weighed about 1.8-3 kg and aged 9 months which were taken from the animal laboratory of Shiraz University of Medical Sciences, Shiraz - Iran. The study was conducted based on the principles of animal rights defined by the Ethic Committee of Shiraz University of Medical Sciences. All animals were housed in individual cages at a constant temperature of 23^o^C in a 12-hour light/dark cycle and feed with a standard pellet diet and filtered water. All procedures were carried out under general anaesthesia using an intramuscular injection of a mixed solution of Ketamine (30 mg/kg) and Thiopental (20 mg/kg). Before beginning the experiment, the rabbits were weighed every week to monitor their health status.

**Retainers**

Forty pairs of maxillary and mandibular central incisors of twenty rabbits were randomly divided into four groups (n= 10/group), as follows: group I was considered as the control and did not receive any orthodontic retainer. Whereas in groups II, III and IV, our experimental groups, Fiber Reinforced Composite (FRC) (Ivoclar-Vivadent; UK), 0.014 inch stainless steel (SS) wire (American Orthodontics-;USA) and 0.175 inch multistrand stainless steel wire (American Orthodontics -;USA) were bonded on labial surfaces of central incisors, respectively. 

To bond the retainers, after cleaning the central incisors with sterile gauze and controlling the moisture with a saliva ejector, the phosphoric acid gel (3M Unitek) was applied to the labial surfaces of incisors for 15-20 seconds for enamel conditioning. Later, the etchant was rinsed off and the teeth were dried thoroughly, and then a thin layer of bonding agent (Ivoclar-Vivadent; UK) was painted over the frosty white enamel surfaces. Retainers were placed on the gingival third of the buccal surface of teeth gently without applying any force. Both ends of the retainers were covered with light-cured composite resins (Ivoclar-Vivadent; UK). The resin bulks were carved with a plastic instrument from the gingival margin to the incisal edge to prevent gingival irritation. After ensuring the absence of any occlusal interference between upper and lower retainers, the composite resins were light cured for 20 seconds (Dentsply; USA).

The plaque and food debris , accumulated around the retainers on the labial surface of the teeth, were cleaned every day by a wet gauze. Since teeth are persistently growing in rabbits, new retainer was replaced immediately in the same day in the instances when a retainer was debonded either by occlusal trauma or by the eruption of incisors. All samples were treated by the same orthodontist using the same technique. 

**Histological** **Examination**

After 60 days of experiment, all the animals were euthanized by injection of high dose of sodium pentobarbital and maxillary and mandibular bones were dissected. Both central incisors and their associated periodontium and supporting bone were sectioned to a 2 cm^2^ thickness and were fixed in 4% paraformaldehyde. Then they were decalcified in 10% Nitric acid for 48 hours, then trimmed and placed in Paraffin blocks. Transverse serial sections (5µm thick) were cut from cervical to apical area and after placing on glass slides, de-paraffinized, hydrated and Hematoxylin-Eosin (H&E) staining was performed. All the slides were viewed with a light microscope (Olympus-BX42, 100X and 400X magnification) in a single blind manner by an experienced oral pathologist.

The infiltration of inflammatory cells in the pulp and periodontium (negative(-), mild(+), moderate(++) and severe(+++)), the new bone formation in the periodontium, the number of osteoblasts and resorptive lacunas in alveolar bone, the mean width of resorptive lacunas' surfaces, the total bone resorptive index (TBRI= multiplying two latter parameters) and the total tooth resorptive index (TTRI), cementum thickness, mean count and diameter of pulp and PDL vessels were measured in 5 separate high power fields of each slide with no overlapping region as quantitative parameters. The orientation and hyalinization of collagen fibers, the congestion in pulp and periodontium and the type of cementum in roots were assessed qualitatively. 

**Statistical** **Analysis**

The histological differences between four groups were evaluated with the Kruskal-Wallis test and pair wise comparisons were carried out by the Mann-Whitney U test. Due to the small sample size, there was no normal distribution in the data, therefore non-parametric analysis was accomplished. A *p* <0.05 was adopted as statistically significant.

## Results

There was no inflammatory cell infiltration in the pulp and periodontal ligament in four groups. New bone formation in supporting tissues was observed more in FRC group but the differences were not statistically significant (*p*= 0.052) There was no significant difference between four groups in terms of mean bone resorption lacunae surface (*p*= 0.286) and TBRI (*p*= 0. 321) and cementum thickness (*p*= 0.739).

There was no root resorption lacuna and TTRI was also equal to zero in all samples. There was significant difference between four groups in terms of osteoblast count (*p*= 0.001). The number of osteoblasts was significantly less in the control group than other groups. There was also a significant difference in terms of number of bone resorption lacunae among four groups (*p*= 0.001). The number of bone resorption lacuna in the control group was significantly less than FRC and 0.014 SS groups (Table 1).

**Table 1:** Percentage of new bone formation, mean of osteoblast count, mean number and surface width of bone resorption lacunae, mean total bone resorption index and mean cementum thickness in four groups. (Control, Fiber Reinforced Composite , 0.014 inch stainless steel wire and 0.175 inch multistrand stainless steel wire).

**Groups**	**Percentage of new bone formation**		**Mean of osteoblast** **Count ± SD**	**Mean number of** **bone resorption** **lacunae ± SD**	**Mean surface width of bone resorption** **Lacunae ± SD (μm)**	**Mean** **TBRI ± SD**	**Mean cementum thickness ± SD (μm)**
	**+**	**++**					
Control	87.5	12.5	6.63±1.061	1.963±0.2825	32.56±6.997	63.74±16.686	6.8913±1.3524
FRC	25.0	75.0	16.75±2.500	2.575±0.1708	28.31±5.414	72.25±9.287	6.3125±0.9437
0.014	100.0	0.0	14.00±1.581	3.060±0.3847	24.90±1.683	76.27±11.458	7.0500±1.1374
0.175	75.0	25.0	13.50±1.291	2.500±0.2582	30.31±5.242	76.38±18.400	6.4625±0.6625

There was significant difference between four groups in terms of the periodontal vessels count (*p*= 0.001). The periodontal vessel count in the control group was significantly lower than FRC and 0.175 MSS groups. The diameter of periodontal vessels was also significantly different in four groups (*p*= 0.001). This diameter in the control group was significantly lower than the other groups. There was significant difference between four groups in terms of pulp vessel count and the diameter of pulp vessels (*p*= 0.002). In control group, these data were significantly more than 0.014 SS and 0.175 MSS groups (Table 2). Two of these latter groups showed hyalinization of the pulp. In all samples the cementum was consisted of acellular extrinsic fibre type and periodontal space and collagen bundles were normal, homogenous and organized. Also in all samples, pulps were congested.

**Table 2:** Mean periodontal ligament vessels count and diameter, Mean pulp vessels count and diameter in four groups. (Control, Fiber Reinforced Composite, 0.014 inch stainless steel wire and 0.175 inch multistrand stainless steel wire)

**Groups**	**Mean PDL vessels** **Count ± SD**	**Mean PDL vessels** **diameter ± SD (μm)**	**Mean pulp vessels** **Count ± SD**	**Mean pulp vessels** **Diameter ± SD (μm)**
Control	8.50 ± 1.773	14.93±3.695	31.00±4.071	18.719±2.932
FRC	14.50± 1.291	41.50±2.131	30.50±2.380	18.813±2.2673
0.014	10.80±0.447	27.20±6.109	3.20±1.483	11.050±1.8742
0.175	13.50±1.291	26.56±3.538	3.50±1.291	10.938±1.2479

## Discussion

Using a fixed retainer made of wire and composite resin and bonded to the lingual tooth aspect is a regular practice after orthodontic treatment [33]. In this study there was no inflammatory cell infiltration in the pulp and periodontal ligament in teeth with the 0.014 inch stainless steel and 0.175 inches multistrand stainless steel wire retainers as well as fiber-reinforced composite retainer. This study found no root resorption and the cementum in all groups consisted of the acellular extrinsic fibre type. The periodontal space and the collagen bundles were normal, homogenous and organized. In line with our results, in other studies, the presence of the retainer and the occasional accumulation of plaque and calculus on the retainer wire, after long-term use, caused no apparent damage to the hard and soft tissues adjacent to the wire [11, 16, 34-35]. Heier et al. [32] reported no significant differences between fixed and removable retainers in neither gingival inflammation nor the accumulation of plaque and calculus after four months. In the study of Pandis et al. no significant difference was found regarding the radiographical bone level between short and long-term usage of lingual wire with fixed retention groups [13]. However, there is some scepticism over the reliability of radiographs in revealing the bone level because of the lack of information on the labial and lingual bony plates [13]. In our histological study, this limitation did not exist and although there was no significant difference in terms of mean bone resorption lacunae surface, number of bone resorption lacuna in control group was significantly less than FRC and 0.014 SS groups. This might be attributed to the limited physiologic tooth movement induced by more rigid fixed retainers like 0.014 SS or FRC. After completion of orthodontic treatment, tooth mobility gradually restored to standard values and bonded lingual retainers were found not impeding this process [36]. An ideal orthodontic fixed retainer should be passive and semi rigid to maintain physiologic tooth mobility after splinting [33]. In our study, FRC and 0.014 SS retainers caused more bone resorption and also the periodontal vessel count and diameter in the control group was significantly lower than FRC and 0.175 MSS groups. It might be concluded that rigidity of FRC can cause detrimental effects on periodontal ligament and sup- porting bone. Schwarze et al. [37] found that even a multistrand wire with a small diameter (0.0155 inch) hindered tooth mobility significantly. Also Watted et al.

found that bonded retainers had a negative impact on on tooth mobility [36]. In our study there was no significant differences between 0.014 inch stainless steel wire and 0.175 inches multistrand stainless steel wire retainers in terms of histological indices. Also in the study of Artun et al., the type of wire used in the retainer did not influence the conditions along the gingival margin, even after a period of wear up to eight years [16]. Zachrisson further recommends using wire diameters that allow for physiologic tooth movement, especially in periodontal high-risk cases [15].

**Figure 1:**
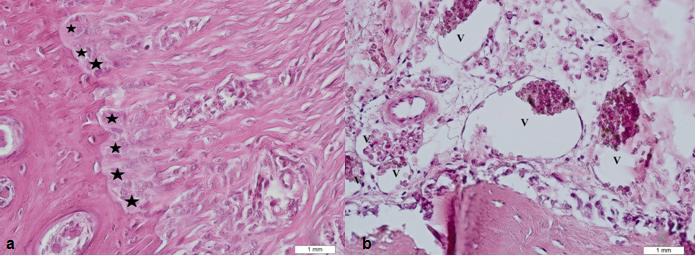
a: Increased bone resorption lacunae tagged by asterics* and b: increased periodontal vessel (V) count in teeth with FRC fixed retainer (H&E staining, 400X)

The physiologic tooth mobility depends mainly on the visco-elastic properties of the periodontal tissues and various individualized anatomic characteristics such as the amount of supporting alveolar bone and the width of the periodontal ligament space [38]. Elastic deflection of the wire component of fixed retainer can occur by a mechanical deformation from masticatory forces [38]. The maximum bite force of male patients on the incisors during biting is 113 N, a force that could cause mechanical deformation of the retainer [39]. A 0.2- mm displacement of the wire exerts a force of 1 N in the vertical axis and about 1.5 N in the horizontal axis [38]. In this study, FRC fixed retainer caused more bone resorption and periodontal vessel count (Figure 1).

**Figure 2:**
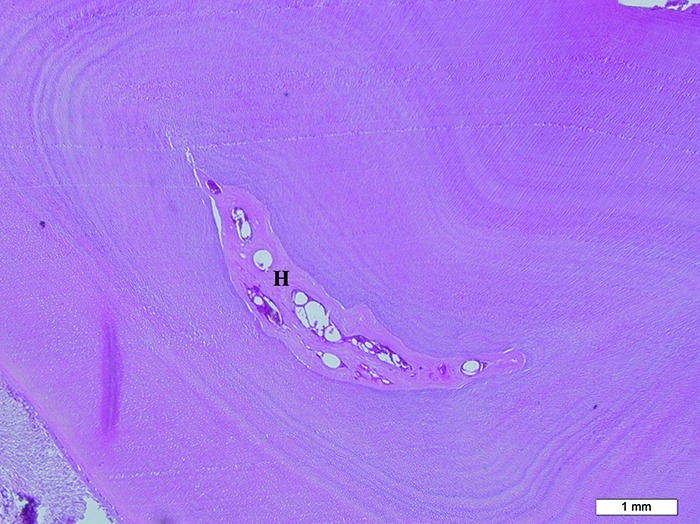
Severe hyalinization (H) of the pulp beside dilation and hyperemia of the vessels in teeth with the 0.014 SS and the  0.175 MSS fixed retainers (H&E staining, 200X)

The pulp vessel count and diameter in the control group were significantly more than the 0.014 SS and the 0.175 MSS fixed retainers. The findings of this study suggest that FRC fixed retainer might cause detrimental effects on periodontal ligament and supporting bone but the 0.014 SS and the 0.175 MSS fixed retainers can cause hyalinization and possibly the necrosis of the pulp (Figure 2). It is desirable for teeth not to be fixed in too rigid position during the orthodontic retention period [14-15, 40-42], but bonded ribbon-resin composite retainer should hold the teeth in a rigid manner [21]. Pandis et al. [13] reported that long-term retention with mandibular bonded appliances resulted in some changes in the periodontal condition of subjects with retainers, which in most cases was confined to a minute increase in various indices and parameters.

In this study the periodontal vessel count and diameter in the control group was significantly lower than other groups. Balenseifen and Madonia [43] found increased number of streptococci and lactobacilli during active treatment with fixed appliances. Similar findings might be true for fixed retainers [44]. In the study of Levin et al. [33] fixed retainers were associated with an increased incidence of recession, plaque retention and bleeding on probing, however, the magnitude of the difference in recession had low clinical significance.

Meng [45] claimed that the increased plaque accumulation is due to the immediate proximity of the wire to the lingual surface, making oral hygiene more difficult. This study reported the retainers as the secondary etiologic factors in the development of gingivitis and periodontitis [45]. More accumulations of debris and a higher prevalence of gingival inflammation have been found in inter-proximal surfaces rather than in lingual areas [46]. Also the proximity of the sublingual salivary ducts increases the risk of calculus formation in the lower incisor region [36]. The multistrand wire entailed the disadvantages of increased plaque accumulation but Artun et al. [11] reported that bonded retainers, made of spiral wire, do not accumulate more plaque and calculus than those made of plain wire. But it must be stated that patients who use a floss-threading device and are aware how to floss between the teeth when wearing a fixed retainer benefit from a good oral hygiene [21]. Zacchrisson emphasized the importance of daily interproximal cleaning with dental floss [14]. If a professional plaque and calculus removal accompanied with oral hygiene instruction is repeated every six months, it is likely that the periodontal health should not be compromised by the presence of bonded lingual wires [32]. In our study the plaque and food debris that accumulated around the retainers on the labial surface of the teeth were cleaned daily by wet gauze, but the oral hygiene was not considered.

If the retainer is placed close to the gingival tissue, it can affect gingival health [33]. It must be emphasized that all the retainers examined in this study were seated meticulously. There was not any contact between inter-dentally papillae and retainer wire in any instance. Also, there was no contact between composite and gingival margin at the bonding sites, and the composite had been trimmed to avoid any retention areas.

An observation period of two months may be too short to draw valid conclusions, especially regarding retainers bonded to the incisors. The findings of this investigation should be interpreted with caution because the duration of this study was not long enough to evaluate long-term effects of FRC retainers on periodontal health. Further investigation could evaluate the long- term histological effects of these retainers. Inter-sample differences might be attributed to the shape of the proximal contacts, varying tooth widths and the position and size of the bonding points [37]. Another limitation of the study is the fact that this experimental study has been done on animals and factors such as the number, shape, and length of the roots, as well as the intrinsic elasticity of the tooth and flow and cleaning

effect of saliva in human would definitely differ from animals’ [47].

In this study periodontal space and collagen bundles were normal and homogenous in all samples. Orthodontic movement to correct tooth rotations is proposed to result in stretching of the collagen fibres [5]. Levin et al. [33] stated that the prevalence of gingival recession was positively correlated with past orthodontic treatment. But in our study the samples had no previous orthodontic treatment. It is suggested that a comparison with an orthodontically tooth movement be attempted in future studies.

## Conclusion

The findings of this study, albeit taking into account the limitations, suggest that fixed retainer caused no root resorption but FRC and 0.014 SS caused more bone resorption. The periodontal vessel count and diameter in the control group was significantly lower than FRC and 0.175 MSS groups. It might be concluded that FRC can cause detrimental effects on periodontal ligament and supporting bone where the 0.014 SS and the 0.175 MSS fixed retainers caused hyalinization and possibly the necrosis of the pulp.
